# How to Achieve the Desired Outcomes of a Complex Intervention in Nursing Homes: A Theory of Change

**DOI:** 10.1093/geroni/igaa057.1234

**Published:** 2020-12-16

**Authors:** Martin Dichter, Jonas Hylla, Almuth Berg, Daniela Eggers, Ralph Möhler, Gabriele Meyer, Sascha Köpke, Margareta Halek

**Affiliations:** 1 University of Cologne, Cologne, Germany; 2 German Center for Neurodegenerative Diseases (DZNE), German Center for Neurodegenerative Diseases (DZNE), Germany; 3 Medical Faculty, Martin Luther University Halle-Wittenberg, Halle, Germany; 4 University of Lübeck, Lübeck, Germany; 5 Bielefeld University, Bielefeld, Germany; 6 Medical Faculty, Halle, Germany; 7 Witten/Herdecke University, Witten, Germany

## Abstract

Background Recent systematic reviews suggest the effectiveness of complex psychosocial interventions to reduce sleep disturbances in people with dementia (PwD) living in nursing homes. However, it is unclear how and under which circumstances these interventions work and which components and processes are crucial determinants for effectiveness. Objectives To develop a Theory of Change (ToC) that describes a causal chain for the reduction of sleep disturbances. Design and Methods The ToC approach is a participatory method in intervention development to generate knowledge about how, why, and under which circumstances interventions are effective. We conducted two expert workshops, a subsequent expert survey (n=12), a systematic literature review, and expert interviews (day and night nurses). Results Necessary preconditions for the reduction of sleep disturbances were identified on staff, management and cultural levels of nursing homes. Intermediate goals like “individual knowledge on PwD is available”, “a specific institutional concept to promote sleep is implemented”, “person-centred care is implemented” and “sleep preferences of PwD are fulfilled” were defined. The intermediate goals, interventions, promoting and inhibiting factors as well as rationales were sorted into a causal chain. All intermediate goals were rated as relevant or highly relevant based on the expert survey. Conclusions The ToC model displays how a complex psychosocial intervention is likely to be effective in reducing sleep disturbances and meeting sleep preferences of PwD in nursing homes. The model is the basis for the development and evaluation of a planned complex psychosocial intervention to prevent and reduce sleep disturbances in PwD.

